# Supplementary material for: Identification, Characterization, and Expression of a β-Galactosidase from Arion Species (Mollusca)
Source: Biomolecules. 2022 Oct 27;12(11):1578. doi: 10.3390/biom12111578 (PMC9687990; doi:10.3390/biom12111578)
Supplement: Supplementary file 1 [file biomolecules-12-01578-s001.zip › biomolecules-1964214-supplementary.pdf]

Table S1. Characteristic peptides from *A. lusitanicus* identified with a LC coupled to an Orbitrap.

Peptide Nr.	Peptide sequence
1	TFEIDYANNTFLK
2	YVSGSMHYSRVPPEYWK
3	TSNTSYLKWVDKWYTELLNK
4	SILGNSTVIYTTDGDGDGYLK
5	YLPLPHGR
6	EYNTSTVLSVPGIRDRAVVMVNNVPIGIIDR
7	IGYGSAMNSNTK
8	GQAIVNNFNIGR
9	GPQVTLYIPQPLFNKPPTANKIYLFELESSPCFES SSQDK

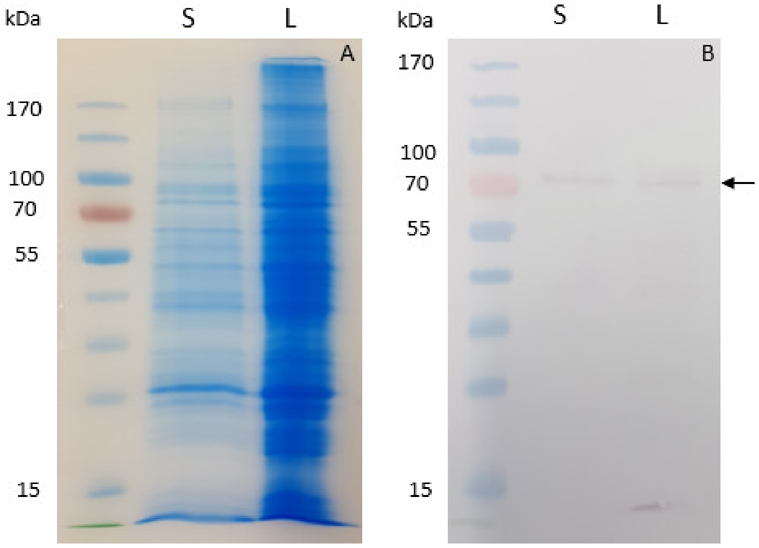

Figure S1. β−Galactosidase expression in Sf9 insect cells (S = supernatant, L = lysate). A) Coomassie blue staining of SDS−Page and B) Western−blot using penta−His monoclonal antibody (1:2500), β−galactosidase ~ 77 kDa. β−Galactosidase is indicated by an arrow.

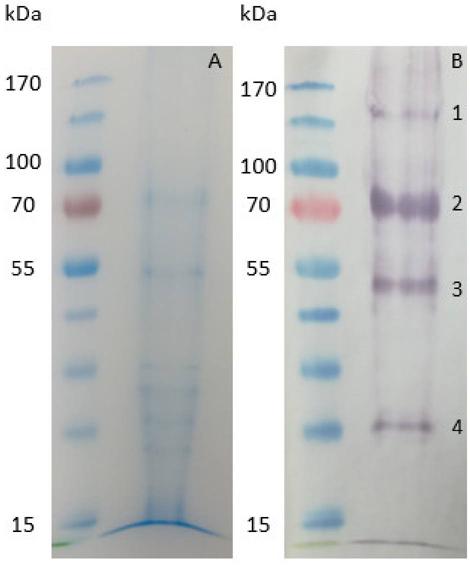

Figure S2. Immunoprecipitation of β−galactosidase from Sf9 lysate fraction. A) Coomassie blue staining of SDS−Page and B) Western−blot using penta−His monoclonal antibody (1:2500). Band Nr. 1 = possible dimeric β−galactosidase structure (~ 154 kDa), Nr. 2 = monomeric β−galactosidase (~ 77 kDa), Nr. 3 = heavy chain of His−tag antibody (~ 50 kDa), Nr. 4 = light chain of His−tag antibody (~ 25 kDa).

Table S2. p−Nitrophenol absorption values (405 nm) of pNP−β−Gal degradation by β−galactosidase from *A. lusitanicus* and *A. vulgaris* at different pH−values. Data points represent values of duplicate measurements (Abs 1 and Abs 2).

pH	Acetat				Citrat				Phosphat				MES				TRIS			
	A. lusitanicus		*A. vulgaris*		*A. lusitanicus*		*A. vulgaris*		*A. lusitanicus*		*A. vulgaris*		*A. lusitanicus*		*A. vulgaris*		*A. lusitanicus*		*A. vulgaris*	
	Abs 1	Abs 2	Abs 1	Abs 2	Abs 1	Abs 2	Abs 1	Abs 2	Abs 1	Abs 2	Abs 1	Abs 2	Abs 1	Abs 2	Abs 1	Abs 2	Abs 1	Abs 2	Abs 1	Abs 2
**3,5**	3,90	4,00	0,12	0,12																
**4**	3,65	3,77	0,12	0,12																
**4,5**	3,22	3,08	0,10	0,10	3,43	3,30	0,13	0,13												
**5**	2,41	2,31	0,08	0,08	2,29	2,17	0,09	0,09												
**5,5**	1,91	2,11	0,07	0,07	1,50	1,45	0,06	0,06					1,50	1,56	0,06	0,06				
**6**					1,14	1,11	0,05	0,05	1,96	1,94	0,07	0,06	0,92	1,04	0,04	0,04				
**6,5**					0,82	0,84	0,04	0,04	1,50	1,50	0,05	0,04	1,33	1,25	0,04	0,04				
**7**									1,10	1,11	0,03	0,03	1,07	1,14	0,03	0,03	1,02	0,87	0,08	0,09
**7,5**									0,94	0,97	0,02	0,02					0,69	0,52	0,05	0,05
**8**																	0,09	0,09	0,02	0,02
**8,5**																	0,12	0,13	0,02	0,02
**9**																	0,10	0,10	0,02	0,02

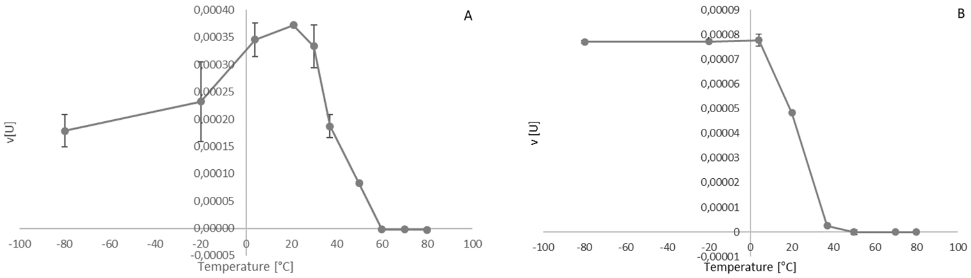

Figure S3. Optimal storage temperature (storage for 16 h at the indicated temperature) of β−galactosidase from (A) *A. lusitanicus* (B) *A. vulgaris*. Data points represent mean values of duplicate measurements in units with corresponding standard deviation. Negative values, due to conversion into units, were set to zero. For experimental details see section 2.6.

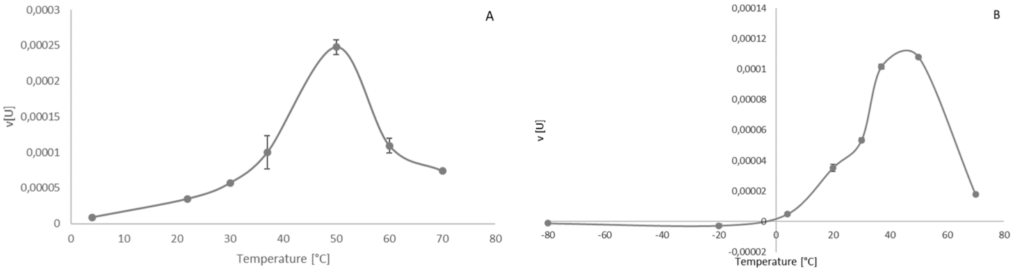

Figure S4. Optimal reaction temperature (reaction assay at the indicated temperature) of β−galactosidase from (A) *A. lusitanicus* (B) *A. vulgaris*. Data points represent mean values of duplicate measurements in units with corresponding standard deviation. For experimental details see section 2.6.

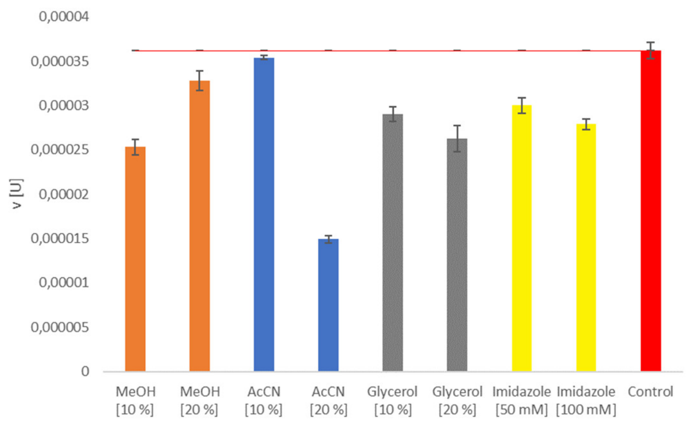

Figure S5. Chemical storage (o/n, RT) of β−galactosidase from *A. vulgaris.* Color code: methanol (orange), glycerol (grey), acetonitrile (blue), and imidazole (yellow). Data points represent mean values of duplicate measurements in units with corresponding standard deviation, and the red line defines the reference line of the control sample. For experimental details see section 2.6.

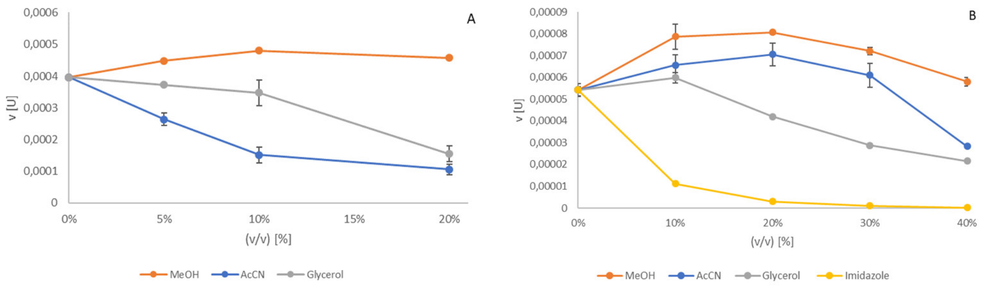

Figure S6. Chemical influence on β−galactosidase during short term incubation (2h, 37 °C) in (A) *A. lusitanicus* and (B) *A. vulgaris.* Color code: methanol (orange), glycerol (grey), acetonitrile (blue), and imidazole (yellow). Data points represent mean values of duplicate measurements in units with corresponding standard deviation. For experimental details see section 2.6.

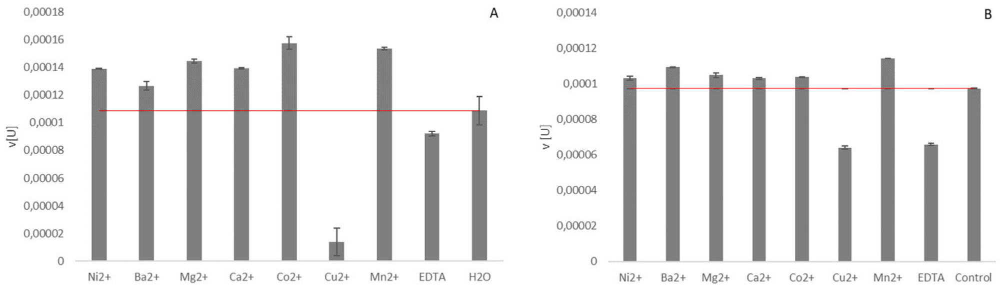

Figure S7. Cation requirement of β−galactosidase from (A) *A. lusitanicus* and (B) *A. vulgaris.* Data points represent mean values of duplicate measurements in units with corresponding standard deviation, and the red line defines the reference line of the control sample. For experimental details see section 2.6.

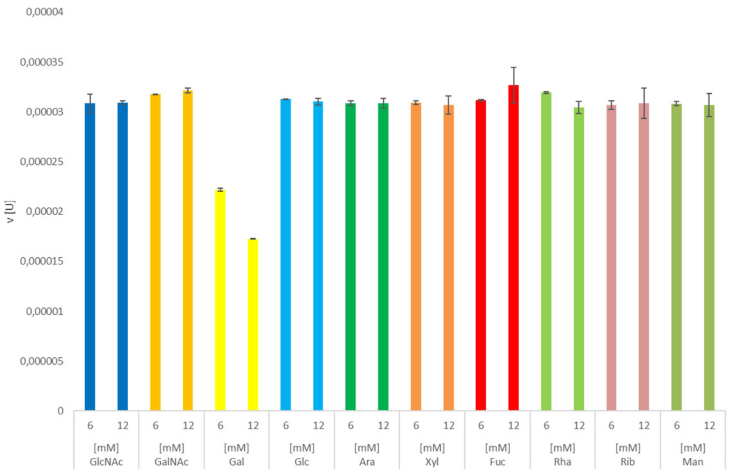

Figure S8. Influence of monosaccharides (6 mM or 12 mM) on β−galactosidase from *A. vulgaris*. Color code: GlcNAc (dark blue), GalNAc (orange), Gal (yellow), Glc (light blue), Ara (dark green), Xyl (gold), Fuc (red), Rha (light green), Rib (rose), and Man (grass−green). Data points represent mean values of duplicate measurements in units with corresponding standard deviation. For experimental details see section 2.6.
